# The value of CT texture analysis in predicting mitotic activity and morphological variants of adrenocortical carcinoma

**DOI:** 10.3389/fradi.2025.1635425

**Published:** 2025-08-07

**Authors:** N. V. Tarbaeva, A. V. Manaev, K. V. Ivashchenko, N. M. Platonova, D. G. Beltsevich, N. V. Pachuashvili, L. S. Urusova, N. G. Mokrysheva

**Affiliations:** ^1^ Endocrinology Research Center, Moscow, Russia; ^2^ Institute for Physics and Engineering in Biomedicine, National Research Nuclear University MEPhI (Moscow Engineering Physics Institute), Moscow, Russia

**Keywords:** сomputed tomography, adrenocortical carcinoma, radiomics, low-grade, high-grade, conventional variant, oncocytic variant, myxoid variant

## Abstract

**Introduction:**

Adrenocortical carcinoma presents significant diagnostic challenges due to its histological heterogeneity and variable clinical behavior. This study aimed to evaluate the diagnostic value of radiomic features in predicting mitotic activity (low/high-grade) and morphological variants (conventional, oncocytic, myxoid) of adrenocortical carcinoma.

**Materials and methods:**

A retrospective analysis of 32 patients with histologically confirmed ACC (18 conventional, 9 oncocytic and 5 myxoid cases) was performed, with mitotic data available for 25 cases (13 low-grade and 12 high-grade cases). Radiomic features including Gray-Level Co-occurrence Matrix (GLCM), Run-Length (GLRLM), Size-Zone (GLSZM), Dependence (GLDM), Neighboring-Tone (NGTDM) and first order features were extracted from four-phase CT using PyRadiomics after manual 3D segmentation. Statistical analysis included Mann–Whitney *U*, Kruskal–Wallis tests, ROC curve (AUC, sensitivity, specificity) and PPV, NPV assessment.

**Results:**

Our analysis demonstrated statistically significant differences between tumor grades with firstorder_Skewness (AUC = 0.924, 95% CI: 0.819–0.986; *p* = 0.005) showing high predictive performance in the venous phase. Radiomic features did not show statistically significant differences between morphological variants of ACC after adjustment for multiple comparisons.

**Conclusion:**

Our results confirm the value of CT radiomics for preoperative stratification of ACC grade, but the question of differentiation of morphological variants remains unresolved and requires further validation in larger cohorts.

## 1 Introduction

The reported incidence of adrenocortical carcinoma (ACC) among adrenal incidentalomas ranges from 0.4% to 4.4% (1). Differential diagnosis of adrenal lesions remains clinically challenging due to tumor heterogeneity and variable clinical presentations. Given the absence of specific symptoms in most patients, imaging plays a pivotal role in accurate diagnosis (2). However, current radiological protocols have inherent limitations in determining the nature of adrenal lesions, and diagnostic accuracy remains heavily dependent on the radiologist's expertise (3). These limitations include insufficient characterization of intratumoral heterogeneity, subjective interpretation of qualitative imaging features, and limited accuracy in indeterminate cases.

Definitive diagnosis of ACC requires histopathological confirmation of adrenocortical origin and assessment of malignant potential, primarily through multiparametric scoring systems and immunohistochemical (IHC) findings (4). Despite standardized diagnostic algorithms and IHC panels, differentiating ACC from benign adrenocortical adenomas remains challenging.

ACCs are classified into four principal histological variants based on their cytomorphological features: conventional, oncocytic, myxoid, and sarcomatoid (4). The conventional variant, the most prevalent, is demonstrates characteristic histopathology, high mitotic activity, and invasive growth into surrounding tissues (5). The oncocytic variant of ACC accounts for 18%–20.5% cases of ACC and is defined by cells with abundant eosinophilic cytoplasm, prominent nuclear polymorphism, and a diffuse growth pattern (6). Emerging evidence suggests this variant may have a more favorable prognosis compared to conventional ACC, especially in cases of low mitotic activity and absence of invasion (7). The myxoid variant, occurring in 12.3%–18% of cases, features variable myxoid stromal components (6, 8) and exhibits aggressive biological behavior due to high metastatic potential and diagnostic ambiguity (6, 8). Adrenocortical sarcomatoid carcinoma, an exceedingly rare and highly aggressive ACC variant, is defined by complete loss of adrenocortical differentiation and prominent sarcomatous morphology (9).

The Weiss scoring system remains the gold standard for evaluating malignant potential in adult adrenocortical lesions (10). However, it demonstrates significant limitations when applied to oncocytic tumors (11), leading to the development of the Lin-Weiss-Bisceglia system for malignancy assessment in these cases (12). Among the Weiss criteria, mitotic count is the only parameter with significant independent prognostic value (13) with a threshold of ≥20 mitoses per 50 high-power fields defining high-grade ACC (4). However, mitotic count reliability is limited by interobserver variability and methodological inconsistencies (14).

Multifactorial assessment has identified independent prognostic factors for ACC, including histological subtype, Ki-67 proliferation index, mitotic activity, and ENSAT tumor stage (6). A novel integrated morphological scoring system was recently introduced to improve prognostic stratification (5). The aggressive biological behavior, poor prognosis, and histopathological heterogeneity of ACC underscore the need for reliable preoperative prognostic biomarkers. These would guide clinical assessment and optimize treatment strategy selection (11).

Modern imaging methods are integral to cancer management, yet their ability to address intratumoral heterogeneity remains limited (15, 16). To overcome the limitations of standard protocols, radiomics has emerged as a comprehensive approach to image analysis. This method involves extraction and analysis of high-dimensional quantitative features from CT images and correlating them with clinical, molecular, and histological data (17).

Studies using CT-texture analysis (CTTA) aim to predict outcomes in various malignancies (18). While CTTA has been explored for differential diagnosis of adrenal lesions, its use in ACC remains limited (19–21). In a study of contrast-enhanced CT (CECT) images from 53 ACC patients radiomic features such as shape flatness, elongation, and grey-level long run emphasis showed statistically significant correlation with Ki-67 expression (*p* = 0.002, 0.01, and 0.04, respectively) (19). Other studies have shown that integrating radiomics with machine learning may further improve existing diagnostic protocols by providing objective quantitative imaging biomarkers (20, 21).

This study aims to advance CTTA application in ACC by evaluating whether radiomic features can distinguish low-grade from high-grade ACC and differentiate major histological variants (conventional, oncocytic, myxoid) based on their radiomic signatures.

## 2 Materials and methods

### 2.1 Study design and cohort

This single-center retrospective uncontrolled cohort study was approved by the Institutional Review Board of the Endocrinology Research Center (Protocol No. 20, dated November 13, 2024). Clinical and imaging data were retrospectively collected from the electronic medical records to include patients meeting the following criteria:

1. Age ≥18 years.
2. Histologically confirmed ACC diagnosis.
3. Surgical resection performed at the Endocrinology Research Center.
4. Availability of preoperative four-phase abdominal CECT (non-contrast, arterial, venous, and delayed phases).
5. Morphological and immunohistochemical verification of ACC subtype.
6. Signed informed consent for the collection and use of biological material.

Exclusion criteria:

1. CT artifacts in the adrenal region (motion artifacts).

### 2.2 CT image acquisition

All patients underwent CECT (non-contrast, arterial, venous, delayed). Some examinations included in the analysis were performed at outside facilities. Studies performed at the Endocrinology Research Center utilized either a 512-slice Revolution CT or 128-slice Optima CT660 scanner (GE Healthcare, USA) with 1.5 mm slice thickness. Contrast administration utilized a MEDRAD Stellant CT Injection System (Bayer, Germany) at 3.5–4 ml/s. Arterial and venous phases were triggered at 10 s and 30 s post-bolus tracking threshold [120 Hounsfield Units (HU) in the aorta at diaphragmatic level]. The delayed phase was acquired 10–15 min post-contrast.

### 2.3 Radiomic feature extraction

#### 2.3.1 Image segmentation

DICOM (Digital Imaging and Communications in Medicine) images were retrieved from the Picture Archiving and Communication System (PACS). The study involved anatomical segmentation of two regions across all CECT phases: (1) the entire adrenal lesion; (2) a fixed-size 3D aortic reference. Manual segmentation was performed by an experienced radiologist with over 5 years of experience using 3D Slicer (v5.6.2).

#### 2.3.2 CT image standardization protocol

Prior to texture analysis, all CT images underwent radio density standardization using the aortic lumen as an internal reference to mitigate interscanner variability in radio density measurements while preserving tissue characteristics. The standardization was performed according to the following equation:

$$I_{\mathrm{standardized}}(x,\;y,\;z)\;=\;\mu_{\mathrm{image}}\;+\;(I(x,\;y,\;z)\;-\mu_{\mathrm{image}})*\frac{\sigma_{\mathrm{ref}}}{\sigma_{\mathrm{aorta}}}$$

where *I*(*x*,*y*,*z*)—original radio density value at voxel (*x*,*y*,*z*);

*μ*_image—the mean radio density value in the region of interest (ROI);
*σ*_aorta—the standard deviation of radio density values in the aortic lumen (internal reference)
*σ*_ref—the reference standard deviation, calculated as the mean *σ*_aorta across the cohort (determined separately for each phase).

#### 2.3.3 Image post-processing and feature extraction

Radiomics features were extracted using PyRadiomics (version 3.1.0) following Image Biomarker Standardization Initiative (IBSI) guidelines. The extracted features included: 18 first-order statistical features, and second-order features including 23 Gray Level Co-occurrence Matrix (GLCM), 16 Gray Level Run Length Matrix (GLRLM), 5 Neighboring Gray Tone Difference Matrix (NGTDM), 14 Gray Level Dependence Matrix (GLDM), and 16 Gray Level Size Zone Matrix (GLSZM) features.

### 2.4 Pathological examination

Tissue samples obtained during surgical treatment were fixed in 10% buffered formalin, processed (Leica ASP6025 S tissue processor, Germany), and paraffin-embedded within 48 h. Paraffin sections (at least 15) with a thickness of 3–4 µm were prepared using a Leica RM 2125 RTS microtome (Leica Biosystems, Germany), placed on polylysine-coated slides (Leica, Germany), and incubated at 37 °C for 12 h. Deparaffinization was performed through series of solutions consisting of three xylenes, two absolute alcohols (80% and 70% alcohol) and distilled water. H&E staining was conducted using a Leica ST5010 AXL stainer (Leica Biosystems, Germany) following standard protocols. ACC diagnosis was confirmed per the 2022 WHO Classification of adrenal cortical tumors (4).

Immunohistochemical analysis was performed on a Leica Bond III automated stainer (Leica Biosystems, Germany) following the manufacturer's standardized protocols. Ki-67 immunostaining was conducted using the MIB-1 clone (1:150 dilution; Dako, Denmark). Slides were digitized using an Aperio AT2 scanner (Leica Biosystems) for further analysis.

The Ki-67 proliferation index was assessed visually in 10 HPFs at ×400 magnification and determined as the percentage of stained nuclei in areas with the highest nuclear staining density (“hot spots”). High proliferative activity was defined as Ki-67 > 10%; low activity as Ki-67 ≤ 10%.

### 2.5 Statistical analysis

Categorical variables were summarized as absolute frequencies and percentages, while continuous variables as median and interquartile range (IQR). Group comparisons were performed using *χ*^2^ tests (categorical), Kruskal–Wallis tests (continuous variables across variants), and Mann–Whitney *U* tests (pairwise comparisons). Feature selection was performed using Spearman's rank correlation analysis, wherein features exhibiting strong collinearity (absolute correlation coefficient >0.9) were identified. Strongly correlated pairs underwent elimination, retaining the variable with higher maximum standardized mean difference (MaxSD), calculated as:

$$\mathrm{MaxSD}\;=\;\frac{\max(\left|\mu_{i}-\mu_{j}\right|)}{\sigma_{\mathrm{pooled}}}$$

*μ*_*i*, *μ*_*j*—mean feature values in subtype/variant *i* and *j*, respectively; *σ*_pooled—pooled standard deviation across all groups.

Further analysis incorporated false discovery rate correction via the Benjamini-Hochberg procedure to account for multiple comparisons. *Post-hoc* pairwise testing was conducted using Dunn's test. Diagnostic performance (sensitivity, specificity, AUC) was evaluated through receiver operating characteristic (ROC) analysis, with optimal cutoffs determined by maximizing Youden's index. Positive/negative predictive values (PPV/NPV) were calculated separately using the optimal cutoffs and cohort prevalence. Bootstrap resampling (1,000 iterations) estimated 95% confidence intervals (CI). Statistical significance was set at *p* < 0.05. Analyses were performed in Python 3.9.21.

## 3 Results

### 3.1 Patient cohort

Among the 32 enrolled patients, 19 (59.4%) presented with right-sided adrenal lesions and 13 (40.6%) with left-sided lesions. Histological assessment of mitotic activity was available for 25 patients, classifying 12 as high-grade ACC and 13 as low-grade ACC. Within this subgroup, patients with high-grade ACC exhibited a significantly higher median age (55 years; IQR: 45–59) compared to low-grade cases (40 years; IQR: 32–40; *p* = 0.021). The low-grade ACC subgroup included 4 males (30.7%) and 9 females (69.3%), while the high-grade subgroup comprised of 3 males (25.0%) and 9 females (75.0%), with no significant sex distribution difference (*p* = 1.000).

Histological subtyping of all 32 cases identified 18 conventional ACCs, 9 oncocytic ACCs, and 5 myxoid ACCs. No statistically significant age differences were noted between variants (*p* = 0.194), though median ages varied: conventional subtype patients were the oldest (56.5 years; IQR: 41.3–59.8), followed by oncocytic (40.0 years; IQR: 40.0–45.0) and myxoid (32.0 years; IQR: 21.0–62.0) subtypes. Sex distribution analysis revealed 6 males (33.3%) and 12 females (66.7%) with conventional ACC, 2 males (22.2%) and 7 females (77.8%) with oncocytic ACC, and 1 male (20.0%) and 4 females (80.0%) with myxoid ACC. Sex distribution did not differ significantly across variants (*p* = 0.756).

### 3.2 Radiomic feature analysis by ACC morphological variants

Texture analysis was performed separately on two volumes of interest (VOI): gross tumor volume (VOIgross-tumour) and functionally active tumor tissue (VOIvital-tumour), excluding areas of necrotis (<30 HU). CT intensity standardization was applied post-segmentation.

#### 3.2.1 VOIgross-tumour analysis

**Figure 1:**
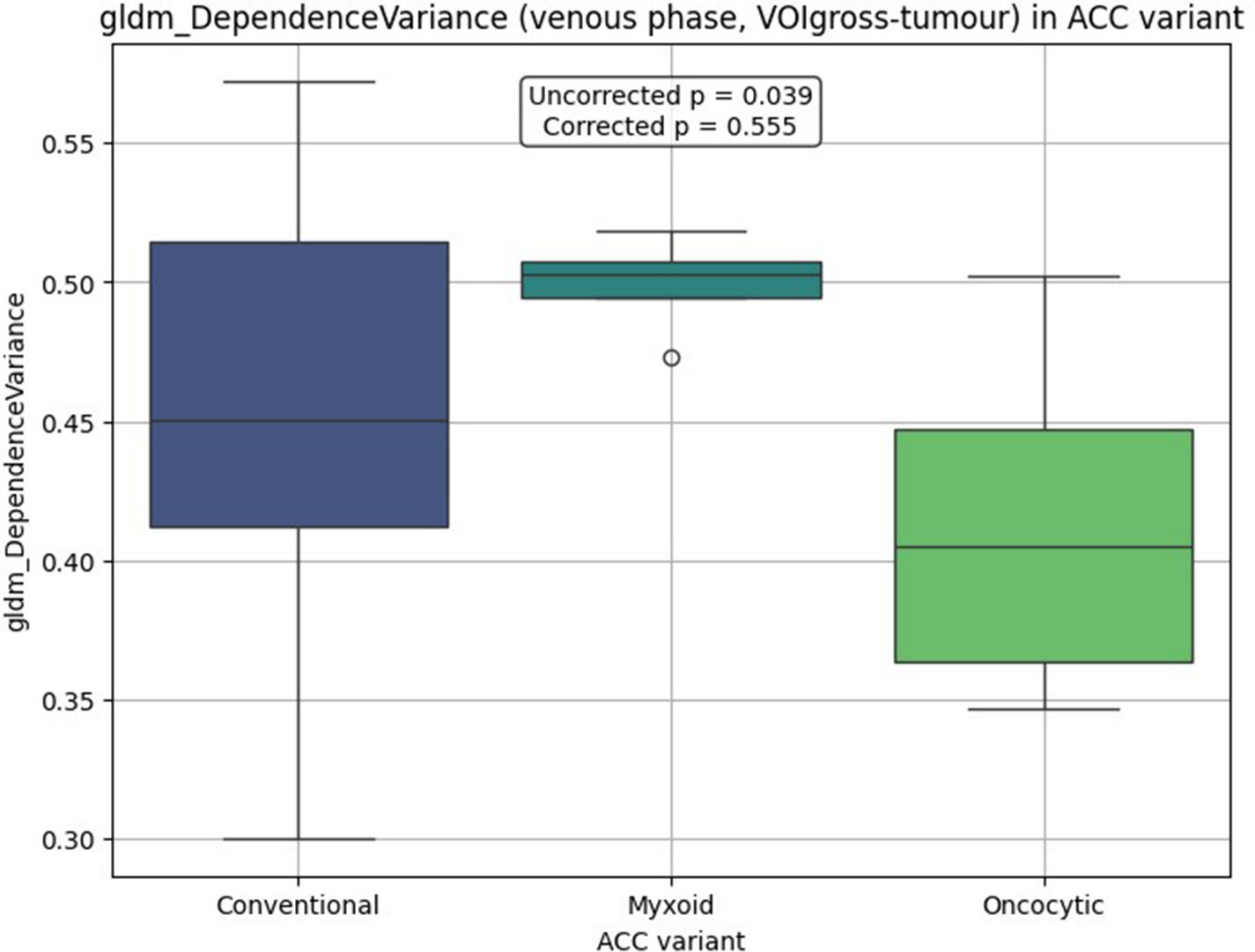
Boxplot distribution by ACC histological variant for gldm_dependenceVariance in VOIgross-tumour analysis.

VOIgross-tumour analysis didn't identify statistically significant differences in radiomic feature between subtypes (Kruskal–Wallis test). Venous phase gldm_DependenceVariance demonstrated the lowest *p*-value (uncorrected *p* = 0.039; corrected *p* = 0.555). Feature distributions are illustrated in Figure 1. *Post-hoc* pairwise comparisons yielded the following results: conventional vs. myxoid (*p* = 0.225), conventional vs. oncocytic (*p* = 0.088), and oncocytic vs. myxoid (*p* = 0.051).

A qualitative trend toward reduced venous phase gldm_DependenceVariance values was observed for the oncocytic variant. Median and interquartile range values of this feature across ACC variants are presented in Table 1.

**Table 1:** The median and interquartile range for venous phase gldm_dependenceVariance in VOIgross-tumour analysis.

Feature	Conventional Me [Q1; Q3]	Myxoid Me [Q1; Q3]	Oncocytic Me [Q1; Q3]
gldm_DependenceVariance	0.451 [0.412; 0.514]	0.503 [0.495; 0.507]	0.405 [0.363; 0.447]

#### 3.2.2 Analysis of VOIvital-tumour (excluding necrosis)

**Figure 2:**
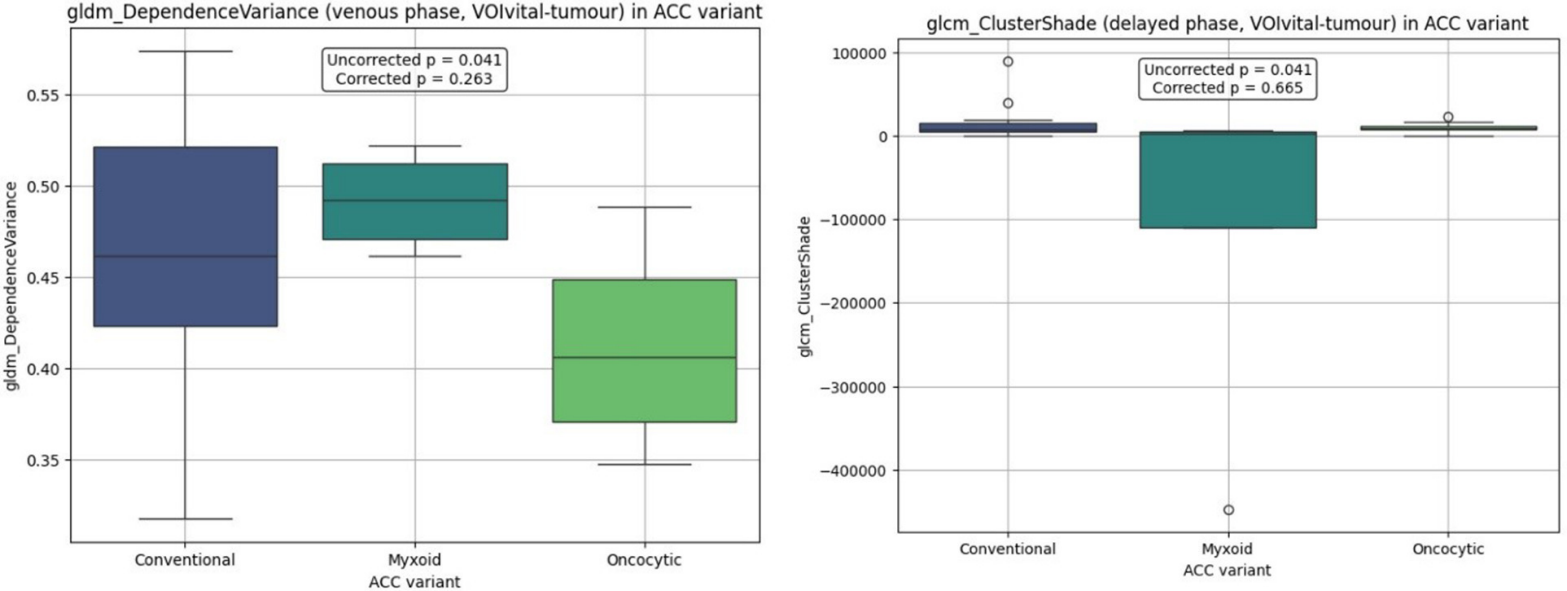
Boxplot distributions by ACC histological variant in VOIvital-tumour analysis.

After excluding necrotic voxels (<30 HU) statistically significant differences weren't identified. The lowest *p*-values were obtained for gldm_DependenceVariance (*p* = 0.263, without correction *p* = 0.041) and delayed phase glcm_ClusterShade (*p* = 0.665, without correction *p* = 0.041). Features distributions are illustrated in Figure 2.

*Post-hoc* analysis revealed the following pairwise comparisons for gldm_DependenceVariance: conventional vs. myxoid (*p* = 0.374), conventional vs. oncocytic (*p* = 0.055), and oncocytic vs. myxoid (*p* = 0.055). For glcm_ClusterShade, results were: conventional vs. myxoid (*p* = 0.042), conventional vs. oncocytic (*p* = 0.585), and oncocytic vs. myxoid (*p* = 0.042). The oncocytic variant demonstrated a tendency toward reduced gldm_DependenceVariance values in the venous phase, while the myxoid variant showed lower glcm_ClusterShade values in the delayed phase. Corresponding median and interquartile ranges across ACC variants are presented in Table 2.

**Table 2:** The median and interquartile range for features in VOIvital-tumour analysis.

Feature	Conventional Me [Q1; Q3]	Myxoid Me [Q1; Q3]	Oncocytic Me [Q1; Q3]
gldm_DependenceVariance	0.462 [0.423; 0.521]	0.492 [0.471; 0.512]	0.406 [0.370; 0.448]
glcm_ClusterShade	7,042.869 [5,055.179; 15,648.898]	2,948.733 [−110,372.848; 4,583.641]	8,195.990 [7,208.781; 11,756.661]

### 3.3 Radiomic feature analysis by ACC histological grade (low-grade and high-grade)

Following the same analytical framework used for radiomic feature analysis by ACC histological subtypes, two approaches were used: gross tumor volume and functionally active tumor tissue analyses.

#### 3.3.1 VOIgross-tumour analysis

**Figure 3:**
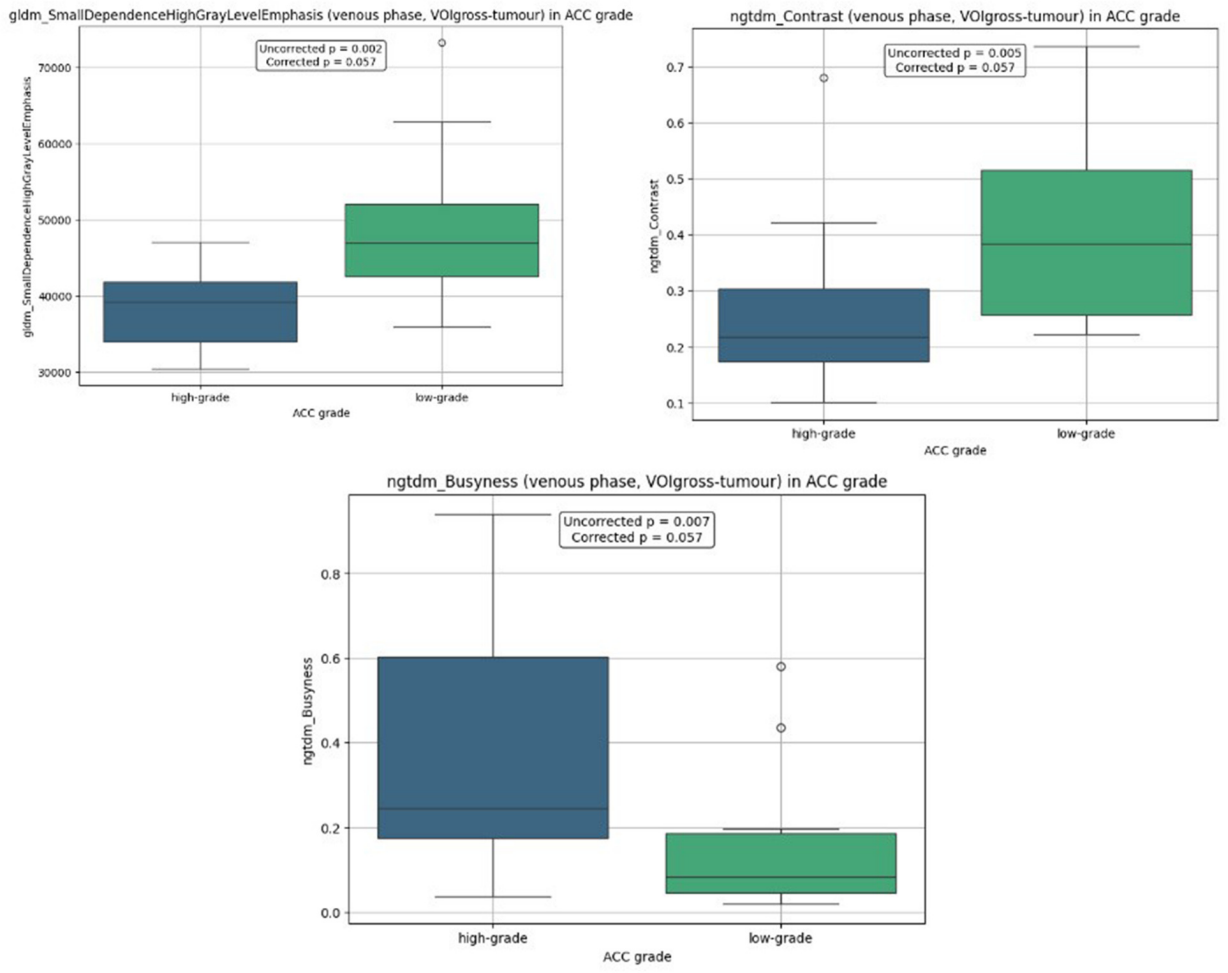
Boxplot distributions by ACC histological grade in VOIgross-tumour analysis.

The analysis didn't identify significant features in non-contrast, arterial, venous, and delayed phases, respectively at *p* < 0.05. Features demonstrating the lowest corrected *p*-values in the venous phase included: gldm_SmallDependenceHighGrayLevelEmphasis (corrected *p* = 0.057; uncorrected *p* = 0.002), ngtdm_Contrast (corrected *p* = 0.057; uncorrected *p* = 0.005), and ngtdm_Busyness (corrected *p* = 0.057; uncorrected *p* = 0.007). Their distributions are presented in Figure 3.

Median and interquartile range values of the features across ACC subtypes are presented in Table 3.

**Table 3:** The median and interquartile range for features in VOIgross-tumour analysis.

Feature	Low-grade Me [Q1; Q3]	High-grade Me [Q1; Q3]
gldm_SmallDependenceHighGrayLevelEmphasis	47,005.011 [42,576.120; 52,077.489]	39,165.277 [33,947.757; 41,806.602]
ngtdm_Contrast	0.383 [0.256; 0.516]	0.217 [0.174; 0.304]
ngtdm_Busyness	0.083 [0.047; 0.186]	0.245 [0.175; 0.602]

#### 3.3.2 Analysis of VOIvital-tumour (excluding necrosis)

**Figure 4:**
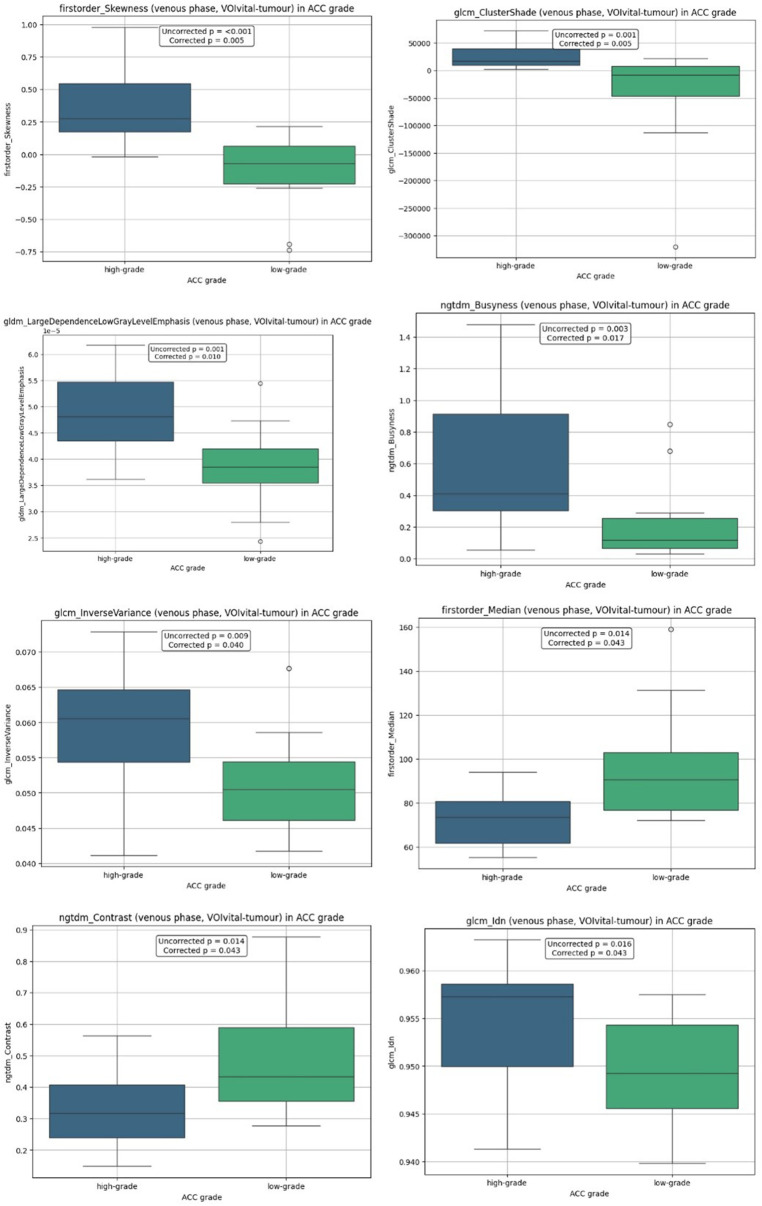
Boxplot distributions by ACC histological grade in VOIvital-tumour analysis.

After excluding necrotic voxels (<30 HU) statistically significant differences were observed for 8 features in the venous phase. Their distributions presented in Figure 4.

Corresponding median and interquartile ranges across ACC subtypes are presented in Table 4.

**Table 4:** The median and interquartile range for features in VOIvital-tumour analysis.

Feature	Low-grade Me [Q1; Q3]	High-grade Me [Q1; Q3]
firstorder_Skewness	−0.072 [−0.227; 0.065]	0.277 [0.173; 0.546]
glcm_ClusterShade	−8,596.004 [−46,162.790; 7,332.865]	17,250.997 [9,718.469; 39,193.306]
gldm_LargeDependenceLowGrayLevelEmphasis	3.845 [3.543; 4.198] ·10-5	4.811 [4.351; 5.471] ·10-5
ngtdm_Busyness	0.115 [0.064; 0.255]	0.408 [0.301; 0.913]
glcm_InverseVariance	0.050 [0.046; 0.054]	0.061 [0.054; 0.065]
firstorder_Median	90.654 [76.900; 102.899]	73.694 [61.880; 80.772]
ngtdm_Contrast	0.433 [0.355; 0.590]	0.316 [0.238; 0.408]
glcm_Idn	0.949 [0.946; 0.954]	0.957 [0.950; 0.959]

The corresponding performance metrics are detailed in Table 5 with high-grade ACC designated as the positive class.

**Table 5:** Diagnostic performance of significant radiomic features and intergroup comparisons of high-grade and low-grade ACC subtypes (VOIvital-tumour).

Feature	AUC (95% CI)	Sensitivity (95% CI)	Specificity (95% CI)	PPV (95% CI)	NPV (95% CI)	Cut-off, high-grade ACC	*p*-value, *U*-test
Venous phase							
firstorder_Skewness	0.924 (0.819–0.986)	0.667 (0.600–1.000)	1.000 (0.643–1.000)	1.000 (0.750–1.000)	0.737 (0.700–1.000)	0.218, >	**0**.**005**
glcm_ClusterShade	0.890 (0.757–0.986)	0.933 (0.600–1.000)	0.714 (0.571–1.000)	0.778 (0.700–1.000)	0.909 (0.700–1.000)	5,523.120, >	**0**.**005**
gldm_LargeDependenceLowGrayLevelEmphasis	0.838 (0.686–0.962)	0.733 (0.533–1.000)	0.857 (0.571–1.000)	0.846 (0.687–1.000)	0.750 (0.650–1.000)	0.0000450, >	**0**.**010**
ngtdm_Busyness	0.814 (0,638–0,957)	0.800 (0,533–1,000)	0.857 (0,643–1,000)	0.857 (0,700–1,000)	0.800 (0,650–1,000)	0.294, >	**0**.**017**
glcm_InverseVariance	0.786 (0.605–0.943)	0.600 (0.467–1.000)	0.929 (0.500–1.000)	0.900 (0,667–1,000)	0.684 (0.619–1.000)	0.059, >	**0**.**040**
firstorder_Median	0.781 (0.595–0.924)	0.933 (0.400–1.000)	0.571 (0.429–1.000)	0.700 (0.650–1.000)	0.889 (0.609–1.000)	85.443, <	**0**.**043**
ngtdm_Contrast	0.767 (0.567–0.905)	0.467 (0.333–1.000)	1.000 (0.357–1.000)	1.000 (0.625–1.000)	0.636 (0.583–1.000)	0.276, <	**0**.**043**
glcm_Idn	0.752 (0.562–0.910)	0.467 (0.333–1.000)	1.000 (0.571–1.000)	1.000 (0.667–1.000)	0.636 (0.583–1.000)	0.958, >	**0**.**043**

Bold values denote statistical significance.

Venous-phase firstorder_Skewness demonstrated both statistical significance (*p* = 0.005) and discriminative capacity (AUC = 0.924, 95% CI: 0.819–0.986). Similarly, glcm_ClusterShade exhibited strong diagnostic utility (AUC = 0.890, 95% CI: 0.757–0.986). Additional radiomic features, including gldm_LargeDependenceLowGrayLevelEmphasis, ngtdm_Busyness, glcm_Idn, firstorder_Median, and glcm_InverseVariance, showed moderate discriminative power (AUC: 0.752–0.838).

## 4 Discussion

In this study, we identified several venous-phase CECT radiomic features with predictive value for differentiating low- and high-grade ACC, including firstorder_Skewness [AUC = 0.924, 95% CI (0.819–0.986)] and glcm_ClusterShade [AUC = 0.890, 95% CI (0.757–0.986)]. However, no significant differences in radiomic features were observed between the major morphological variants of ACC, likely due to the limited cohort size.

Radiomics is a promising method for identifying image-based biomarkers in adrenal lesions; however, its diagnostic utility in ACC remains underexplored. To our knowledge, no published studies have evaluated diagnostic utility of radiomic features in across ACC histological variants/subtypes.

The study implemented CT image post-processing for variance standardization, addressing well documented challenges in radiomics reproducibility (20). Post-processing enhances the potential reproducibility of results and accounts for variations in texture features across different CT scanners.

Texture analysis of functionally active tumor tissue demonstrated diagnostic utility for several venous-phase radiomic features in discriminating low-grade and high-grade ACC: firstorder_Skewness, glcm_ClusterShade, gldm_LargeDependenceLowGrayLevelEmphasis, ngtdm_Busyness, glcm_InverseVariance, firstorder_Median, ngtdm_Contrast, and glcm_Idn.

Low-grade ACC exhibited distinct radiomic characteristics: predominance sub-median intensity values (negative firstorder_Skewness values); local transitions indicative of hypodense regions (predominantly negative glcm_ClusterShade values); sparse large homogeneous low-density zones (low gldm_LargeDependenceLowGrayLevelEmphasis values); relatively smooth texture intensity transitions (low ngtdm_Busyness); pronounced local heterogeneity and inter-voxel contrast (low glcm_InverseVariance and glcm_Idn values); and notable macro-scale contrast (high ngtdm_Contrast values). Collectively, these findings suggest a tumor architecture characterized by abundant microheterogeneity and discrete density variations, potentially reflecting rich microcapillary networks and compartmentalized perfusion. The co-occurrence of microheterogeneity (low glcm_InverseVariance, glcm_Idn) macro-contrast (high ngtdm_Contrast) likely reflects from interlaced microvascular structures and fibrotic septa generating local density fluctuations, while lacking extensive low density fields.

High-grade ACC exhibited distinct radiomic signatures characterized by: predominance of high intensity values (positive firstorder_Skewness) and local transitions (predominantly positive glcm_ClusterShade values) likely reflecting exclusion of necrotic areas from the analysis; large homogeneous hypodense zones, potentially indicating incomplete exclusion of necrotic areas by the 30 HU threshold (elevated gldm_LargeDependenceLowGrayLevelEmphasis values); less gradual texture intensity transitions (elevated ngtdm_Busyness) combined with reduced local microheterogeneity and inter-voxel contrast (high glcm_InverseVariance and glcm_Idn values), suggesting either presence of extensive homogeneous cellular regions or residual necrotic tissue; diminished large scale contrast (low ngtdm_Contrast values). Collectively, these findings indicate that high-grade ACC is characterized by large homogeneous zones (probably necrotic) with reduced macrotextural heterogeneity but greater microstructural disorganization. Notably, high-grade ACCs exhibited lower median attenuation values of solid components (low firstorder_Median) compared to low-grade tumors, which possibly reflects the higher proportion of remaining necrotic areas in high-grade ACC.

Assessment of clinical relevance and effect magnitude yielded valuable insights. Venous-phase firstorder_Skewness and glcm_ClusterShade demonstrated both high statistical significance and exceptional discriminative capacity, establishing its potential as an imaging biomarker for high mitotic activity. Other features with significant differences showed moderate discriminative power, yet their structural interpretations revealed clinically meaningful distinctions in tumor architecture as previously detailed.

The statistically significant corrected *p*-values obtained in our study indicate that excluding voxels with attenuation values below 30 HU yields superior diagnostic performance compared to whole-tumor analysis. This enhancement likely stems from the restricted analytical focus, which improves detection of tumor aggression-associated patterns—specifically, local heterogeneity, asymmetry and textural complexity. Inclusion of low-density regions in the analysis may introduce confounding effects from necrotic components, thereby obscuring critical microstructural features.

Our analysis revealed no significant discriminative power for distinguishing ACC morphological variants in either VOIgross-tumour or VOIvital-tumour analyses, a limitation attributable to the cohort size. Within VOIvital-tumour analysis venous phase gldm_DependenceVariance demonstrated the strongest statistical trend (*p* = 0.041 uncorrected; *p* = 0.263). *Post-hoc* testing revealed borderline significance (*p* = 0.055) for distinguishing the oncocytic variant from both conventional and myxoid. Oncocytic ACC demonstrated reduced spatial heterogeneity in homogeneous regions (lower gldm_DependenceVariance values). This radiomic pattern aligns with hallmark histological uniformity of oncocytic ACC characterized by predominantly of large polygonal cells with abundant, granular, eosinophilic cytoplasm that form compact homogeneous cell nests, fundamentally distinguishing the oncocytic variant from both conventional and myxoid ACC subtypes at the microstructural level (22).

Integration of radiomic signatures into clinical practice holds potential to enhance preoperative risk stratification for ACC. Early identification of high-grade tumors optimize surgical planning, intensify surveillance protocols, or facilitate timely initiation of mitotane-based adjuvant therapy (8). Nevertheless, clinical implementation faces significant barriers, including the absence of standardized radiomics processing frameworks and limited clinician awareness of quantitative imaging biomarkers.

The present study has several limitations, primarily its cohort size (*n* = 32) and retrospective single-center design, which collectively limit generalizability and elevate overfitting risks in high-dimensional radiomic analysis (23). While feature pre-selection and multiple hypothesis correction were implemented, statistically significant differences may reflect cohort specific patterns rather than generalizable biological characteristics. Features exhibiting exceptionally high predictive accuracy in small samples should be interpreted with particular caution, as such performance likely reflects overfitting to data “noise” rather than clinically relevant signatures. These constraints are exemplified by the inability to establish definitive subtype-specific texture patterns for ACC morphological variants, where observed trends failed to reach statistical significance (*p* > 0.05).

Expanding the dataset could improve the statistical significance of the observed patterns and enable modeling of complex data relationships. For instance, a prior radiomic-based deep learning study analyzing 794 adrenal lesions achieved high diagnostic accuracy (AUC = 0.974, sensitivity = 92.7%, specificity = 92.8%) in predicting the presence of malignant adrenal masses, though performance varied across subtypes (18). Therefore, further validation through larger, prospective multicenter studies is necessary to confirm these findings and develop reliable predictive models. Validation across diverse populations requires addressing protocol standardization. While we mitigated scanner variability using aortic-reference standardization, future multicenter studies should evaluate this method's generalizability.

In addition, further development of this methodology requires investigation of additional factors, such as the impact of contrast enhancement parameters, ROI segmentation variability, and integration of radiomics with established biomarkers like genetic and immunohistochemical tumor profiles in order to improve diagnostic accuracy and optimize risk stratification strategies.

## 5 Conclusion

This study demonstrates that CT texture analysis of functionally active tumor tissue, particularly utilizing venous phase radiomic features, showed predictive value in differentiating high-grade from low-grade ACC based on mitotic activity, highlighting its potential diagnostic value for preoperative assessment. However, radiomic features did not significantly differentiate between ACC morphological variants (conventional, oncocytic, myxoid). The successful implementation of image variance standardization underscores the importance of post-processing for enhancing diagnostic feature reproducibility. While promising, these findings require validation in larger, prospective multicenter cohorts.

## Data Availability

The raw data supporting the conclusions of this article will be made available by the authors, without undue reservation.
