# Supplementary material for: The value of CT texture analysis in predicting mitotic activity and morphological variants of adrenocortical carcinoma
Source: Front Radiol. 2025 Aug 7;5:1635425. doi: 10.3389/fradi.2025.1635425 (PMC12367671; doi:10.3389/fradi.2025.1635425)
Supplement: Supplementary file 1 [file Table1.docx]

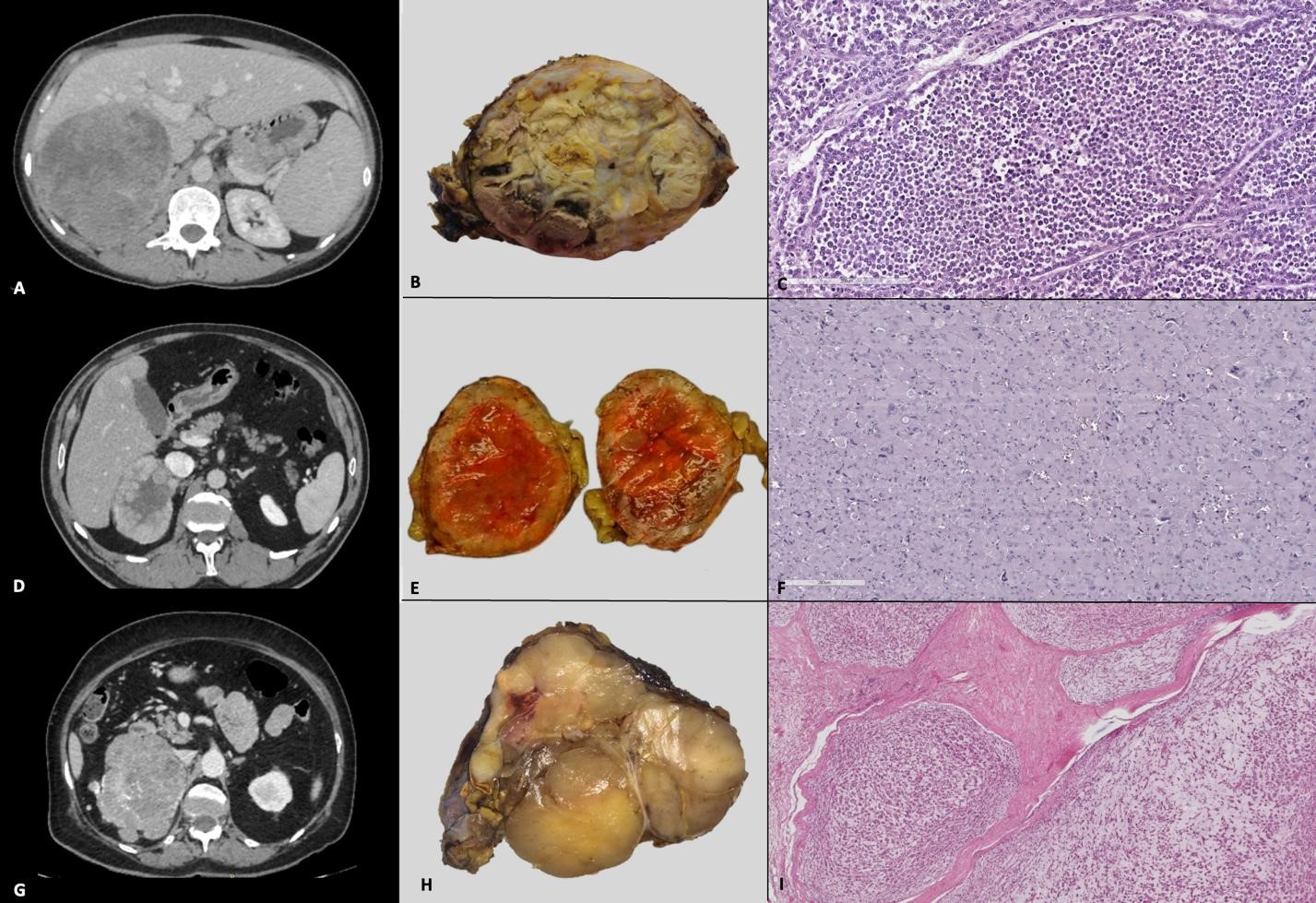


Representative imaging and histopathological features:

A – CECT (venous phase), conventional ACC variant).

B – Macroscopic appearance of conventional ACC.

C – Microscopic appearance of conventional ACC: tumor composed of small to medium polymorphic cells forming solid structures (hematoxylin and eosin (**H&E**) stain, ×100).

D – CECT (venous phase), oncocytic ACC variant.

E – Macroscopic appearance of oncocytic ACC.
